# Development and initial validation of the Influences on Patient Safety Behaviours Questionnaire

**DOI:** 10.1186/1748-5908-8-81

**Published:** 2013-07-29

**Authors:** Natalie Taylor, Sahdia Parveen, Victoria Robins, Beverley Slater, Rebecca Lawton

**Affiliations:** 1 Bradford Institute for Health Research, Bradford Royal Infirmary, Duckworth Lane, Bradford BD9 6RJ, United Kingdom; 2 Australian Institute of Health Innovation, Faculty of Medicine, University of New South Wales, Sydney NSW 2051, Australia; 3 Institute of Psychological Sciences, University of Leeds, Leeds LS2 9JT, United Kingdom

**Keywords:** Barriers, Behavior change, Theoretical domains framework, Healthcare, Patient safety

## Abstract

**Background:**

Understanding the factors that make it more or less likely that healthcare practitioners (HCPs) will perform certain patient safety behaviors is important in developing effective intervention strategies. A questionnaire to identify determinants of HCP patient safety behaviors does not currently exist. This study reports the development and initial validation of the Influences on Patient Safety Behaviors Questionnaire (IPSBQ) based on the Theoretical Domains Framework.

**Methods:**

Two hundred and thirty-three HCPs from three acute National Health Service Hospital Trusts in the United Kingdom completed the 34-item measure focusing on one specific patient safety behavior (using pH as the first line method for checking the position of a nasogastric tube). Confirmatory factor analysis (CFA) was undertaken to generate the model of best fit.

**Results:**

The final questionnaire consisted of 11 factors and 23 items, and CFA produced a reasonable fit: *χ*^*2*^ (175) = 345.7, *p* < 0.001; CMIN/DF = 1.98; GFI = 0.90 and RMSEA = 0.06, as well as adequate levels of discriminant validity, and internal consistency (*r* = 0.21 to 0.64).

**Conclusions:**

A reliable and valid theoretically underpinned measure of determinants of HCP patient safety behavior has been developed. The criterion validity of the measure is still unknown and further work is necessary to confirm the reliability and validity of this measure for other patient safety behaviors.

## Background

There is substantial evidence of unsafe care in health systems globally [1-3]. Harms resulting from unsafe care include infections, incorrect medicines or procedures, missed or delayed diagnosis, falls, and are often preventable [4]. Understanding the factors that influence those behaviors associated with patient safety is an important first step in the development of strategies to improve care [5]. However, interventions to improve patient safety have typically been developed intuitively and have relied on managers or other experts using strategies such as education, persuasion, or reminding people to change their behavior [6], rather than adopting a more theoretical approach to understanding and addressing key barriers and levers to behavior change [7]. Although there is some support for the effectiveness of the aforementioned strategies [8], evidence suggests there are more and less appropriate times to use particular intervention techniques depending on the specific factors (*e*.*g*., motivation, confidence, environment, emotion) affecting behavior change [7,9]. However, individuals tasked with designing and implementing behavior change interventions may find it difficult to choose from the abundance of health behavior theories, which are often insufficiently specified to determine when or how to modify factors that are to be targeted through an intervention [10].

The theoretical domains framework (TDF) was developed using an expert consensus and validation process to rationalize and reconceptualize the theoretical constructs from psychological and organizational theory that influence behavior and behavior change [9]. The framework was developed to make a plethora of behavior change theories more accessible for interdisciplinary audiences involved in implementation, and can be used to understand the barriers and levers to change in a range of contexts [11,12]. Researchers applying the TDF to healthcare practitioner (HCP) behavior have, to date, relied on qualitative interviews to understand the factors influencing HCP behavior change [5,13]. Although interviews are useful for gaining a detailed understanding of barriers and levers to change, they are resource intensive, time consuming, and often allow for a small sample size, limiting generalizability of findings across, for example, a hospital Trust. A questionnaire based on the TDF, on the other hand, might be a quicker way to identify key domains of behavior change among a larger sample. A questionnaire approach also has the potential to be used in practice by HCPs, improvement teams, or others who have been tasked with facilitating behavior change in their organization. For these groups, the knowledge, skills, and time required to use qualitative interviews might be prohibitive.

Although the TDF has been used to develop a handful of questionnaires examining HCP barriers and levers to working with patients to improve health behaviors such as smoking [14-16], to our knowledge, a questionnaire to understand the factors affecting healthcare practitioner behavior change for patient safety does not exist in the literature. Furthermore, although there are other validated patient safety questionnaires e.g., [17], these tend to measure general attitudes, culture, and climate within a particular ward or organization, rather than to understand barriers to performing a specific patient safety behavior. Given the extensive range of behaviors associated with ensuring the safety of patients, it was deemed necessary to address this gap in the literature and develop a measure that accounts for this factor. Therefore, this study reports on the development and initial validation of the Influences on Patient Safety Behaviors Questionnaire (IPSBQ).

## Methods

### Context

Between April 2011 and September 2012, the Yorkshire and the Humber Health Innovation and Education Cluster (HIEC: http://yhhiec.org.uk/themes/patient-safety) Patient Safety Theme worked with three hospitals to support the implementation of a National Patient Safety Agency (NPSA) Alert aimed at ‘reducing the harm caused by misplaced nasogastric (NG) feeding tubes’ [18]. The IPSBQ was developed and tested as part of this work.

Misplacement of NG tubes is not uncommon and can have serious consequences. Between 2005 and 2011, there have been 21 deaths and 79 cases of harm in the United Kingdom (UK) due to feeding into the lungs through misplaced NG feeding tubes. Although there is no completely failsafe method for checking the placement of the NG tube, one of the recommendations is that the first line method for confirming tube position should be to check the pH of the aspirate from the stomach. If the pH is >5.5, or it is not possible to obtain aspirate, it is only then appropriate to send for an X-ray to check the position of the tube [18]. The position of the NG tube is not always clear from the X-ray, and therefore the possibility of errors of interpretation is high.

### Identifying a target behavior

The NG tubes NPSA alert sets out to reduce the risk of feeding into the lungs, rather than the stomach. It was necessary to identify which behavior change was central to producing this patient safety improvement from the range of recommendations provided in the guideline. Establishing a specific target behavior is an important aspect of the TDF approach to behavior change because this framework relies on the detailed identification of the barriers affecting one specific behavior, rather than a set of behaviors [9].

To identify the target behavior, informal discussions with front line National Health Service (NHS) staff were followed by audits in each Trust to assess current practice for inserting and checking NG tube position. As a result, the target behavior was confirmed as ‘using pH the first line method for checking the position of an NG tube’.

### Development of the IPSBQ

**Figure 1:**
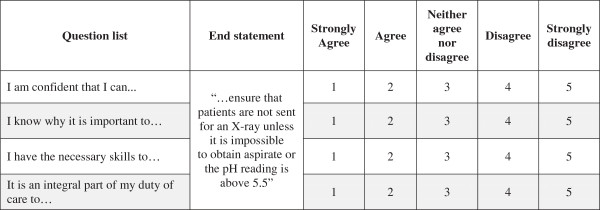
Influences of Patient Safety Behaviours Questionnaire excerpt with example target behaviour (nasogastric tubes).

The 34-item IPSBQ was based on the TDF [9], which specifies 12 domains of behavior change: knowledge, skills, social/professional role and identify, beliefs about capabilities, beliefs about consequences, motivation and goals, memory attention and decision processes, environmental context and resources, social influences, emotion, behavioral regulation, and nature of the behavior.^a^ Although the current study focuses on establishing the reliability and validity of the IPSBQ in relation to NG tubes behavior, the items were developed in the context of this alert and three additional areas of patient safety (midazolam overdose, injectable medicines, and medicines reconciliation) to ensure the questionnaire would be applicable for a range of target behaviors. To maximise construct validity, items were developed using a combination of: evidence from work which has previously used the TDF framework to understand or change behavior in healthcare settings [11,13], and meetings with 16 HCPs (members of implementation teams we formed as part of a wider project to work with hospital Trusts to implement patient safety guidelines, consisting of nurses, junior doctors, registrars, consultants, and pharmacists) regarding their experiences of adopting one of the four aforementioned patient safety behaviors. Three questionnaire items were developed for each of the 11 domains (with the exception of ‘knowledge’ for which four items were developed) and designed to be applicable to a range of patient safety behaviors (Figure 1). The items were both positively and negatively phrased and participants were asked to rate their level of agreement with each statement on a five-point likert scale (1 = strongly agree; 5 = strongly disagree); negative items were reverse scored. A higher mean score on a domain indicates a stronger barrier to behavior change for participants. A team consisting of clinical (VR) and behavior change specialists (NT, RL) provided detailed feedback on the comprehension, face validity, and the item fit within a domain. A convenience sample of 15 HCPs (junior doctors and nurses) then commented on this draft and final amendments were made.

### Participants and procedure

Consultation with the local NHS ethics committee indicated that ethical approval was not required for this work because this project was a service evaluation focusing on NHS staff. Data was collected between June 2011 and June 2012 across three hospital Trusts focusing on the NG tubes alert, using two approaches to participant recruitment. First, clinical teams involved in the wider project were encouraged to distribute hard copies of the questionnaire to colleagues involved in the key behaviors. Second, a link to an online version of the questionnaire was made available to ward managers and post-graduate medical education administrators for dissemination to staff. Recruitment strategies targeted all staff for whom the key behavior was relevant (doctors, nurses, and dieticians) on wards where NG tubes were used. Although responsibility for ensuring the correct positioning of the NG tube varied, junior doctors across all three Trusts were primarily responsible for ensuring an NG tube is in the correct position before commencing (or giving permission to commence) a feed. With this in mind, we planned to over-represent junior doctors within the sample. Participants provided details of their hospital and ward location, job role, number of years’ since graduation, number of years in current organization, date of birth, and the last three letters of postcode; no personally identifiable information was provided. Participants were informed that the questionnaire would take no more than five minutes to complete, and that their responses would be confidential and anonymous. No incentive was offered for completion.

### Data analysis

In the present study, a theoretical framework was being tested so it was deemed appropriate to use confirmatory factor analysis (CFA) as a model generating tool [19] to establish the initial construct validity of the IPSBQ, and to remove items to reduce participant burden when completing the measure. Sample size guidance indicated that 200 to 300 participants would be adequate for CFA analysis [20,21].

Data were input into SPSSv19 and screened prior to imputing missing values using expectation maximization [22]. CFA was used to test an 11-factor model using maximum likelihood estimation. Guidelines for testing model fit followed guidance by Hooper [23]: a chi square to degrees of freedom ratio (CMIN/DF) of less than 2.00, the goodness of fit index (GFI) ≥0.90, and the root mean square error of approximation (RMSEA) ≥0.05. Post-hoc analysis was used to improve the model fit [19,24] by inspecting modification indices (MIs) [25], standardized residuals (SRs) [26,27], and item content.^b^ Discriminant validity was assessed using Fornell and Larkner’s tests [28-30].^c^ Inter-item correlations were used to test for internal consistency, with values above 0.15 to 0.50 being the optimal range [29,30]. Although we could not directly test criterion validity of the questionnaire using correlation because we measured compliance at Trust level rather than individual self-reported behavior, we assessed an indicator of criterion validity by comparing mean total IPSBQ scores against audited compliance rates for the use of pH to check the first line method of tube position in each Trust.

## Results

### Identifying a target behavior

A range of behaviors were identified as possible areas to target based on objective audit evidence (*e*.*g*., ensuring pH of aspirate was checked prior to every feed following initial confirmation of tube placement), but each team decided that targeting the use of pH as the first line method for checking tube position was the most important part of the process to target.

### Descriptive statistics

The final sample consisted of 233 healthcare professionals who completed the IPSBQ that examined perceived barriers to using pH as the first line method for checking the placement of NG tubes. One hundred and twenty-seven (55.4%) participants were junior doctors or registrars, 27 (11.6%) were consultants, 42 (18%) were nurses, 7 (3%) were dieticians, and 28 (12%) did not provide details regarding their profession. The average age of the sample was 34.06 years (*SD* = 9.23). On average, participants had been qualified for 8.1 years (*SD* = 9.1), and had worked for their particular Trust for 4.5 years (*SD* = 7.3).

Missing value analysis indicated that data was missing completely at random (*χ*^2^ (1418) = 1512, *p* = 0.04), therefore missing values were imputed. The descriptive statistics pertaining to scores for each domain (post CFA) are presented in Table 1. Mean scores for each domain ranged from 2.09-2.87. On average, the strongest barrier to performing the target behavior (checking pH first line) across all three hospitals was reported as ‘skills’, followed by ‘beliefs about capabilities’, ‘motivation and goals’, ‘social influences’, and ‘environmental context and resources’.

**Table 1:** Descriptive statistics and internal consistency scores for the 11 TDF domains

**Barrier**	**Mean (SD) post CFA *n *= *233***	**Inter-item correlation post CFA**
Knowledge	2.29 (0.92)	0.64
Skills	2.87 (0.98)	0.62
Social and professional identity	2.10 (0.77)	0.23
Beliefs about capabilities	2.74 (0.92)	0.43
Beliefs about consequences	2.09 (0.77)	0.45
Motivation and goals	2.70 (0.80)	0.21
Cognitive processes, memory and decision making	2.59 (0.80)	0.23
Environmental context and resources	2.63 (0.77)	0.47
Social influences	2.68 (0.74)	0.22
Emotion	2.17 (0.84)	0.62
Action Planning	2.21 (0.73)	0.43

### Construct validity of the IPSBQ

The initial CFA showed data did not fit the model well (*χ*^2^ (472) = 1304.0, *p* <0.001; CMIN/DF = 2.76; GFI = 0.71 and RMSEA = 0.09), thus post hoc model fitting was conducted. This resulted in the removal of 11 items: two from the knowledge domain, one item each from nine other domains, and no items from the environmental context and resources domain on the basis of large MIs (above 10), and SRs > ±2.58, and assessment of item content (Additional file 1). This revised model (containing 23 items) was found to fit the data satisfactorily (*χ*^2^ (175) = 345.7, *p* <0.001; CMIN/DF = 1.98; GFI = 0.90 and RMSEA = 0.06).

### Discriminant validity

All 11 domains were found to display discriminant validity according to Fornell and Larkner (1981), suggesting that each domain measures a distinct construct.

### Internal consistency

The inter item correlations ranged from 0.21 to 0.64, with the domains professional identity, motivation and goals, memory, attention and decision making, and social influences demonstrating adequate levels of internal consistency (see Table 1).

### Indicator of criterion validity

The mean total IPSBQ scores and rates of compliance for the use of pH as the first line method to check tube position across each Trust are presented in Table 2. The difference in total barriers scores between Trusts was small (range = 23.8 to 25.1), and this was reflected in the similar levels of compliance (range = 12 to 20%). Results indicate that higher barriers were reported for those Trusts that demonstrated a lower level of compliance for the use of pH first line.

**Table 2:** Total barriers score and rates of compliance with target behavior across each trust

**Measures**	**Trust A**	**Trust B**	**Trust C**
Mean total barriers score (SD)	23.8 (5.8)	24.6 (5.8)	25.1 (4.8)
pH method used first line to check tube position (number of notes audited)	20% (49)	14% (44)	12% (43)

## Discussion

Based on a theoretical framework of behavior change, 11 scales measuring the psychosocial domains of patient safety behavior change among HCPs were developed and tested. With the exception of Taylor, Lawton, and Conner [31], to our knowledge, this is first time that CFA has been used to validate the TDF domains, which resulted in good construct validity, discriminant validity, and internal consistency.

This is the first study to develop a measure of the barriers to practitioner behavior change for patient safety (a full version of the IPSBQ can be found in Additional file 2), the novel design of which allows for application to a range of patient safety behaviors. Following further testing and confirmation of its validity, the IPSBQ may be used to identify barriers across a large sample—this information might be complemented by a smaller sample of focus groups to cross validate, and further understand the details about, the key barriers identified. The IPSBQ could potentially act as a tool for developing theoretically underpinned large-scale interventions or, at a local level, for working with staff to co-develop realistic and feasible strategies to address key barriers. The latter approach may be especially relevant if there are differences in key barriers to behavior change between or within organizations, because this would allow for tailoring of interventions for specific contexts.

Despite establishing initial reliability and validity of the IPSBQ, a number of limitations should be noted. First, although the sample size was adequate, a larger sample would allow for increased confidence in the reliability of the measure. Second, we did not directly ask participants about the regularity with which they checked the position of NG tubes, so were unable to apply a filter to assess differences in barriers according to the level of performance of the target behavior. Third, the RMSEA index of fit result did not meet agreed standards for construct validity [23]; however there are variations for optimal levels in the literature, for example 0.06 has been suggested as the ideal maximum [24], but 0.08 is also considered an acceptable upper limit [32]. The weak RMSEA index may be partly related to the attempt to ensure each domain contained two items in order to reduce the participant burden of completion. Even so, several measures reduced to two items per domain have demonstrated valid and reliable properties, and been successfully used in healthcare research [33]. Furthermore, some domains (*e*.*g*., beliefs about consequences, social influences) are represented by a large number of constructs, which might be viewed as relevant to more than one domain (*e*.*g*., the ‘anticipated regret’ construct can be found in both the beliefs about consequences and emotion domains; the ‘social/group norms’ construct can be found in both the social influences and social and professional role and identity domains); this makes it difficult to both generate a questionnaire that represents all of the constructs, does not take a considerable amount of time for participants to complete, and which demonstrates good discriminant validity. We have worked with each of these constraints to produce the first validated version of this measure. For example, we worked with clinicians in the development of all of the items for each domain, and consulted the interview questions from the TDF [9], literature regarding particular domains (*e*.*g*., Theory of Planned Behavior for motivation and goals, beliefs about capabilities, social influences, beliefs about consequences), and work which has recently been undertaken to develop measures based on the TDF e.g., [11,31]. Nonetheless, these points highlight the need for additional work to improve the reliability and validity of the tool, and also demonstrate some of the implications that operationalizing heterogeneous domains into a parsimonious questionnaire can have for selecting some items and omitting others. Fourth, this study has not confirmed the criterion validity of the IPSBQ on patient safety behavior. Although these early results indicate that the IPSBQ can detect higher reported barriers for individuals within Trusts demonstrating lower compliance with the target patient safety behavior, further work is needed to establish whether this measure can explicitly demonstrate criterion validity. In the first instance, this could be achieved by adding a self-report measure of behavior to the questionnaire.

The NG tubes alert provides 17 recommendations for NHS organizations/individuals to achieve, many of which involve a range of behaviors. This can make it difficult for HCPs to define an appropriate target behavior to address, because it requires consideration of compliance—(identifying which elements of current practice did not meet the recommendations [34]), specificity (focusing on a specific behavior that it is possible to change following identification of associated barriers [9]), and impact (defining a behavior that is likely to have most effects on outcomes [35]). During this work, other possible key behaviors were identified due to low compliance, but teams decided that targeting the use of pH as the first line method for checking tube position was the crucial aspect of the process. This was because teams recognized that in addition to low compliance, changing this behavior had the potential to not only prevent the need for X-ray (and therefore reduce the risk of misinterpretation), but also to improve the chances of pH being used for checking tube position for subsequent feeds, thus reducing the need for multiple X-rays and further risk of misinterpretation (impact). Nonetheless, this highlights the complexities associated with defining a specific target behavior for change, especially if attempting to use a description of the behavior in relation to a set of questionnaire items that relate to 11 domains of behavior change. Future work with the TDF in the context of patient safety should investigate how appropriate it is to select a single behavior if clinicians are performing multiple behaviors, or whether it is possible to operationalize the TDF to elicit behavior change when multiple behaviors are targeted.

While a questionnaire of this kind might be a useful method for identifying the relative strength of barriers, the absolute strength of a barrier is more difficult to measure. None of the domains assessed in this questionnaire had a mean score above the mid-point of the scale, despite compliance with this guideline being low according to case note audit results. This might imply a tendency for people to underestimate the barriers to behavior change, or alternatively an affinity to respond in a socially desirable way. Future work might assess the impact of using a four-point Likert scale on reporting of barriers, because evidence has demonstrated that this can reduce social desirability response bias [36]. The potential for this measure to be used or adapted to identify levers to support behavior change might also be an area worth investigating.

We have presented an indicator of criterion validity by demonstrating that the total barriers score for each Trust increases as compliance with the target behavior decreases. However, a sum score may not be entirely appropriate because this indicates that all domains are equivalent proximal predictors of behavior. Nevertheless, although many of the constructs from within different domains stem from complex mediating processes from the theories from which they are sourced and are arguably inter-related, to our knowledge this has not yet been tested. Therefore, the current results perhaps provide a basis or rationale for future research to examine the extent to which each domain (and which of the associated constructs) predicts behavior in the context of patient safety.

In addition to undertaking further work to test the 23-item measure, and improve the reliability, validity, and generalization of the IPSBQ, the next stage of this research should also aim to establish whether the key barriers identified by this measure can be targeted with theoretically underpinned and pragmatic interventions. Following this, the impact of these interventions on changing healthcare practitioner behaviors, as well as their reported barriers [37], should be tested. Given the IPSBQ has been used to identify the factors affecting change for one patient safety behavior, work should also be undertaken to understand if the measure can be used to reliably identify barriers to other target behaviors; this next phase is currently underway in areas relating to patient safety and midazolam, gentamicin, and medicines reconciliation. Finally, since the development of the IPSBQ, an updated version of the TDF has been published [38]; this includes three new domains (optimism, goals, and reinforcement) and revisions to some of the constructs associated with each domain. Therefore, the new aspects of the TDF will need to be considered for inclusion in a revised measure.

## Conclusion

The IPSBQ can be used by researchers and practitioners working in areas of healthcare improvement, implementation, and patient safety. Further research should be undertaken to fully understand the uses and limitations of the measure, but initial results suggest that it demonstrates reliable and valid properties for assessing the psychosocial factors affecting practitioner behavior change. These findings provide sufficient support to suggest that this measure can be used to identify barriers to behavior change among healthcare staff; the next stage should be to discover if this measure can be used as a tool for informing the development of theoretically informed tailored interventions. It is recommended that the IPSBQ be used in future research to understand whether targeting key domains with matched interventions can change practitioner behaviors for patient safety.

### Availability of supporting data

The data set(s) supporting the results of this article are available from the first author.

## Endnotes

^a^The ‘nature of the behavior’ determinant was, as in the Michie et al. (2005) paper, accorded a different order to the rest, as it describes the dependent variable, which in this case is ‘using pH as the first line method to check tube position’. It is therefore not treated as a domain of behavior change, but its constructs (such as habit, stages of change, and representation of tasks) were considered throughout the development of the questionnaire in relation to the target behavior.

^b^MIs were provided by AMOS for all parameters constrained to zero and indicate when an item may cross load or load onto a different factor [20]. The standardised residual matrix identifies pairs of items that are either under or over-predicted by the model [21], for which values > +/−2.58 are considered to be large [22].

^c^Two constructs display discriminate validity if the average of the estimate of variance extracted exceeds the square of the correlation between the two latent constructs, and the confidence interval around the correlation estimate between the two factors includes 1.0. Inter-item correlations were used to test for internal consistency, with values above 0.15-0.50 being the optimal range [23-25]. Full workings out and results for discriminant validity are available from the author.

## Abbreviations

IPSBQ: Influences on Patient Safety Behaviors Questionnaire; CFA: Confirmatory factor analysis; RMSEA: Root mean square error of approximation; CMIN/DIF: Chi square to degrees of freedom ratio; GFI: Goodness of fit index; TDF: Theoretical Domains Framework; HIEC: Health Innovation and Education Cluster; NPSA: National Patient Safety Agency; NG: Nasogastric; MIs: modification indices; SRs: Standardised residuals.

## Competing interests

The authors declare that they have no competing interests.

## Authors’ contributions

NT led the design and coordination of the study, performed the statistical analysis, and led the writing process. SP performed the statistical analysis, and helped to draft the manuscript. VR advised on questionnaire item content, and helped to draft the manuscript. BS participated in the design of the study, and helped to draft the manuscript. RL participated in the design of the study, advised on questionnaire item content, and helped to draft the manuscript. All authors read and approved the final manuscript.

## Authors’ information

NT and RL have previously worked on projects that involve using the TDF framework to identify barriers and design interventions using theoretically underpinned behavior change techniques to design tailored interventions to address key barriers for a range of health behaviors. SP is a health psychologist specialising in socio-cognitive influences on stress and risk behaviors; we drew on her statistical expertise for this project. VR is a renal registrar and provided a crucial insight into the nature of clinical work, which influenced the content of the questionnaire. BS is an organizational psychologist who has drawn upon her knowledge and experience of implementation science to contribute to this work. The papers that follow this manuscript are currently in preparation and demonstrate the use of the questionnaire as a tool to identify key barriers, and the effect of interventions tailored to key barriers to performing specific professional patient safety behaviors.

## Supplementary Material

Additional file 1 **Item removal details.** Table A. Items retained and removed from each domain, Table B. Statistical and theoretical justifications for item removal. Click here for file

Additional file 2 Influences on Patient Safety Behaviors Questionnaire following initial validation. Click here for file
