# Supplementary material for: Development and initial validation of the Influences on Patient Safety Behaviours Questionnaire
Source: Implement Sci. 2013 Jul 29;8:81. doi: 10.1186/1748-5908-8-81 (PMC3846501; doi:10.1186/1748-5908-8-81)
Supplement: Additional file 1 — Item removal details. Table A. Items retained and removed from each domain, Table B. Statistical and theoretical justifications for item removal. [file 1748-5908-8-81-S1.doc]

| Determinant area | Items removed (item label) | Items retained |
| --- | --- | --- |
| Knowledge | - I know why it is important to…(know1) - I do not know where to find information to help…(know4) | - I know what the NPSA guidelines say about the need to…(know2) - I fully agree with the NPSA guidelines which instruct staff to…(know3) |
| Skills | - I have the necessary skills to…(skills1) | - Training is not offered to me regularly enough to...(skills2) - Training is not adequate to...(skills3) |
| Social and professional identity | - It is an integral part of my duty of care to…(profid1) | - It isn’t my responsibility to…(profid2) - I am clear about what my role should be in the process to…(profid3) |
| Beliefs about capabilities | - I am confident that I can...(cap1) | - I do not find it easy to…(cap2) - I have previously encountered problems when trying to…(cap3) |
| Beliefs about consequences | - The costs outweigh the benefits of trying to…(conseq1) | - It does not matter too much if I do not...(conseq2) - It will be bad for the patient if I do not...(conseq3) |
| Motivation and goals | - I intend to...(mg2) | - Emergencies and other priorities get in the way of me being able to...(mg1) - Other guidelines conflict with trying to…(mg3) |
| Memory, attention and decision making | - I often forget to...(cog1) | - I habitually (or usually)…(cog2) - There are justifiable reasons for why I often decide not to…(cog3) |
| Environmental context and resources | - No items removed | - There is not a good enough system in place to…(enviro1) - I have the necessary resources (e.g., correct/enough equipment, staff, etc.) (enviro2) - Verbal and written communication between staff is clear enough for me to...(enviro3) |
| Social influences | - Other staff encourage me to…(socinfl1) | - Other staff don’t seem to…(socinfl2) - My superiors would like me to…(socinfl3) |
| Emotion | - I feel frustrated when I do not...(emo3) | - I feel anxious if I think about having to...(emo1) - I worry if I think about having to...(emo2) |
| Action Planning | - I always plan how I will...(ap1) | - Plans in my head often get muddled when trying to...(ap2) - Things are too unpredictable to make plans to...(ap3) |

Additional file 1. Item removal details

Table A. Items retained and removed from each domain

Table B. Statistical and theoretical justifications for item removal

| Model statistics | MI data* | SR data | Notes |
| --- | --- | --- | --- |
| CMIN/DF = 2.760, GFI = 0.706,  RMSEA = 0.087, Chi sq =1304.0, df =472 | Know4 6 MIs >10  Know4 with skills3 = 21.26 | 3 SRs > +/- 2.58 | Training likely to be associated with provision of information; remove know4 |
| CMIN/DF = 2.650, GFI = 0.730,  RMSEA = 0.084, Chi sq =1166.1, df =440 | Know1 6 MIs >10  Know1 with profid1 = 22.93 | 6 SRs > +/- 2.58 | Knowing a behaviour is important may be related to feeling it’s a duty; remove know1 |
| CMIN/DF = 2.594, GFI = 0.745  RMSEA = 0.083, Chi sq =1060.8, df =409 | Skills1 6 MIs >10  Skills1 with cap1 = 36.74 | 5 SRs > +/- 2.58 | Believing one possesses necessary skills likely to be associated with confidence; remove skills1 |
| CMIN/DF = 2.437, GFI = 0.776  RMSEA = 0.079, Chi sq =923.7, df =379 | Ap1 5 MIs >10  Ap1 with cog2 = 14.70 | 8 SRs > +/- 2.58 | Those who usually performs a behaviour may be more likely to because they make plans; remove ap1 |
| CMIN/DF = 2.352, GFI = 0.797  RMSEA = 0.076, Chi sq =823.3, df =350 | Mg2 2 MIs >10  Mg2 with cog2 = 19.35 | 2 SRs > +/- 2.58 | Those who usually perform a behaviour may have stronger intentions to do that behaviour; remove mg2 |
| CMIN/DF = 2.255, GFI = 0.820  RMSEA = 0.074, Chi sq =726.0, df =322 | Cap1 1 MI >10  Cap1 with profid1 = 21.00 | 1 SR > +/- 2.58 | It may be that those lacking in confidence did not believe it was part of their duty of care; remove cap1 |
| CMIN/DF = 2.20, GFI = 0.836  RMSEA = 0.07, Chi sq =649.5, df =295 | Conseq1 2 MIs >10  Conseq1 with mg1 = 21.45 | 4 SRs > +/- 2.58 | Wording was perceived as generally confusing as to whether it was positive or negative; remove conseq1 |
| CMIN/DF = 2.159, GFI = 0.846  RMSEA = 0.071, Chi sq =580.8, df =269 | Socinfl1 2 MIs >10  Socinfl1 with emo2 = 10.23 | 2 SRs > +/- 2.58 | Those encouraged/asked to do the behaviour may be worried about having to do it; remove socinfl1 |
| CMIN/DF = 2.042, GFI = 0.858  RMSEA = 0.067, Chi sq =498.2, df =244 | No MIs >10 | 1 SR > +/- 2.58 | Use of brackets and two different words may have made the item difficult to understand; remove cog1 |
| CMIN/DF = 2.011, GFI = 0.869  RMSEA = 0.066, Chi sq =442.5, df =220 | No MIs >10 | 2 SRs > +/- 2.58 | Use of the word integral may have been confusing for some participants; remove profid1 |
| CMIN/DF = 1.992, GFI = 0.879  RMSEA = 0.065, Chi sq =392.4, df =197 | No MIs >10 | 1 SR > +/- 2.58 | Item phrasing confusing as difficult to interpret whether it is positive or negative; remove emo3 |
| CMIN/DF = 1.976, GFI = 0.889  RMSEA = 0.065, Chi sq =345.7, df =175 | No MIs >10 | 0 SRs > +/- 2.58 | No items to remove |

*Full item content for each item label can be found in Table A
