# Supplementary material for: Development and initial validation of the Influences on Patient Safety Behaviours Questionnaire
Source: Implement Sci. 2013 Jul 29;8:81. doi: 10.1186/1748-5908-8-81 (PMC3846501; doi:10.1186/1748-5908-8-81)
Supplement: Additional file 2 — Influences on Patient Safety Behaviors Questionnaire following initial validation. [file 1748-5908-8-81-S2.docx]

**Staff experiences of working with nasogastric tubes**

**Thank you** for taking the time to complete this questionnaire, which aims to gather perspectives from the sharp end of patient care before working alongside staff to create hospital innovations. It is not a test of your knowledge, but a way for us to find out some of the barriers staff face with certain hospital practices, so please answer the questions as honestly as you can. First, please complete the following information:

| Unique identifier: | DOB: ………/………/………. Last 3 digits of postcode: ………….. …………… ………….. |
| --- | --- |
| Name of hospital: |  |
| Speciality/work area: |  |
| Profession (please circle) | Consultant Doctor Nurse Other |
| No. of years in speciality/work area | Years: …………………….. Months: ………………………………… |
| No. of years since graduation: | Years: …………………….. Months: ………………………………… |
| How long at this Trust: | Years: …………………….. Months: ………………………………… |

This questionnaire is designed to ask you about your experiences with nasogastric tubes and should take no more than **3 minutes** to complete. The Leeds hospital Trust policy guidelines for inserting nasogastric feeding tubes indicate that:

- pH testing is the **first line test** method, with between 1 and 5 as the safe range, and that each test and test result is documented on a chart at the patient’s bedside
- X-ray is **only** used as a second test when no aspirate can be obtained or the pH indicator paper has failed to confirm the position of the tube

| **Yes** | **No** |
| --- | --- |

Please indicate (circle) if you were **already aware** of the information stated in the bullet points above:

**In accordance with the guideline, each phrase on the questionnaire is followed by the end statement:**

*“…ensure that patients are only sent for an X-ray if it is impossible to obtain aspirate or the pH reading is above 5.5”*

When rating your level of agreement with each phrase, please think about **all the things that might affect you being able to** ensure that patients are only sent for an X-ray if it is impossible to obtain aspirate or the pH reading is above 5

**Please indicate your level of agreement with the following statements:**

| **Question list** | **End statement** | **Strongly**  **Agree** | **Agree** | **Neither agree nor disagree** | **Disagree** | **Strongly**  **disagree** |
| --- | --- | --- | --- | --- | --- | --- |
| 1. Emergencies and other priorities get in the way of me being able to... | …ensure that patients are only sent for an X-ray if it is impossible  to obtain aspirate or the pH reading is above 5.5 | 1 | 2 | 3 | 4 | 5 |
| 1. There is not a good enough system in place to… |  | 1 | 2 | 3 | 4 | 5 |
| 1. I feel anxious if I think about having to... |  | 1 | 2 | 3 | 4 | 5 |
| 1. I know what the NPSA guidelines say about the need to… |  | 1 | 2 | 3 | 4 | 5 |
| 1. I fully agree with the NPSA guidelines which instruct staff to… |  | 1 | 2 | 3 | 4 | 5 |
| 1. Training is not offered to me regularly enough to... |  | 1 | 2 | 3 | 4 | 5 |
| 1. It isn’t my responsibility to… |  | 1 | 2 | 3 | 4 | 5 |
| 1. I do not find it easy to… |  | 1 | 2 | 3 | 4 | 5 |
| 1. It does not matter too much if I do not... |  | 1 | 2 | 3 | 4 | 5 |
| 1. I habitually (or usually)… |  | 1 | 2 | 3 | 4 | 5 |
| **Question list** | **End statement** | **Strongly**  **Agree** | **Agree** | **Neither agree nor disagree** | **Disagree** | **Strongly**  **disagree** |
| 1. I have the necessary resources (e.g., correct/enough equipment, staff, etc.) | …ensure that patients are only sent for an X-ray if it is impossible  to obtain aspirate or the pH reading is above 5.5 | 1 | 2 | 3 | 4 | 5 |
| 1. Other staff don’t seem to… |  | 1 | 2 | 3 | 4 | 5 |
| 1. I worry if I think about having to... |  | 1 | 2 | 3 | 4 | 5 |
| 1. Plans in my head often get muddled when trying to... |  | 1 | 2 | 3 | 4 | 5 |
| 1. Training is not adequate to... |  | 1 | 2 | 3 | 4 | 5 |
| 1. I am clear about what my role should be in the process to… |  | 1 | 2 | 3 | 4 | 5 |
| 1. I have previously encountered problems when trying to… |  | 1 | 2 | 3 | 4 | 5 |
| 1. It will be bad for the patient if I do not... |  | 1 | 2 | 3 | 4 | 5 |
| 1. Other guidelines conflict with trying to… |  | 1 | 2 | 3 | 4 | 5 |
| 1. There are justifiable reasons for why I often decide not to… |  | 1 | 2 | 3 | 4 | 5 |
| 1. Verbal and written communication between staff is clear enough for me to... |  | 1 | 2 | 3 | 4 | 5 |
| 1. My superiors would like me to… |  | 1 | 2 | 3 | 4 | 5 |
| 1. Things are too unpredictable to make plans to... |  | 1 | 2 | 3 | 4 | 5 |
